# CORRELATION OF OSTEONECROSIS RATES IN THE SURGICAL TREATMENT OF PROXIMAL HUMERAL FRACTURES ACCORDING TO THE NEER AND HERTEL CLASSIFICATIONS

**DOI:** 10.1590/1413-785220233103e268183

**Published:** 2023-07-17

**Authors:** FABIO FABIAN BUSCARIOLO, IGOR ARTHUR COSTA PARRON, ELZIR FINIZOLA COSTA, MARCOS VINICIUS FELIX SANTANA, EDUARDO MISAO NISHIMURA, EIFFEL TSUYOSHI DOBASHI

**Affiliations:** 1 Rede D’Or Sao Luiz, Hospital IFOR, Serviço de Ortopedia e Traumatologia, São Bernardo do Campo, SP, Brazil.; 2 Sociedade Brasileira de Cirurgia do Ombro e Cotovelo, São Paulo, SP, Brazil.; 3 Universidade Federal de Sao Paulo, Escola Paulista de Medicina, Departamento de Ortopedia e Traumatologia, São Paulo, SP, Brazil.

**Keywords:** Humeral Head, Classification, Osteonecrosis, Prognosis, Humeral Fractures, Cabeça do Úmero, Classificação, Osteonecrose, Prognóstico, Fraturas do Úmero

## Abstract

**Objective:**

To predict the risk of osteonecrosis (ON) according to the Neer and Hertel et al. classification for surgically treated proximal humeral fractures after at least one year of follow-up.

**Methods:**

This is a retrospective, cross-sectional, and observational cohort study. A total of 44 patients, 16 (36.36%) men and 28 (63.63%) women, with a mean age of 61.36 years, participated in this study. Lesions were categorized according to Neer and Hertel’s classifications, considering the preoperative prognosis for ON. After at least a year of follow-up, patients were reassessed. Data were evaluated using IBM SPSS Statistics^®^.

**Results:**

A total of three patients (6.8%) developed osteonecrosis. Comparisons showed no statistically significant difference, but we observed a superior association of osteonecrosis for the Hertel classification than that of Neer.

**Conclusion:**

Both classifications showed a similar ability to identify patients at low risk of developing ON. New studies with a greater number of participants and sample homogeneity may intensify the value of the evaluation of clinical applicability and predictive capacity of the studied classifications with greater significance and correlation. ***Level of Evidence III, Case Control Study.***

## RESUMO

**Objetivo:**

Prognosticar o risco de osteonecrose (ON), segundo os critérios de Hertel et al. e a classificação de Neer, para fraturas do úmero proximal tratadas cirurgicamente após, pelo menos, um ano de seguimento.

**Métodos:**

Estudo de coorte retrospectiva, transversal e observacional. Foram encontrados 44 pacientes, sendo 16 (36,36%) do sexo masculino e 28 (63,63%) do feminino, com média de idade de 61,36 anos. As lesões foram categorizadas de acordo com os critérios de Neer e de Hertel et al., considerando o prognóstico pré-operatório para ON. Após pelo menos um ano de seguimento, os pacientes foram reavaliados. Os dados foram analisados por meio do programa IBM SPSS Statistics.

**Resultados:**

Três pacientes (6,8%) evoluíram com ON. As comparações não demonstraram diferença estatística significante, embora tenha sido possível observar associação superior para a classificação de Hertel et al. em comparação com a de Neer.

**Conclusão:**

As duas classificações apresentaram habilidade semelhante para identificar pacientes com baixo risco de desenvolver ON. Sugere-se a realização de novos estudos com maior número de participantes e homogeneidade da amostra para intensificar o valor da avaliação da aplicabilidade clínica e da capacidade preditiva das classificações estudadas, com aumento da significância e da correlação. **Nível de Evidência III, Estudo Caso Controle.**

## INTRODUCTION

Proximal humeral fractures account for about 4 to 5% of all injuries affecting the mature skeleton. It is the second most frequent when we specifically consider upper limb trauma. In women, due to physiological osteopenia determined by natural aging, its incidence is twice as large as that of men.^1^

Osteoporosis is directly related to the incidence of these lesions, which are becoming more common due to the increasing number of older adults in our population. Such a condition makes it difficult to fix bone damage, especially with conventional plates and screws. ^(^ ^1^ ^),(^ ^2^ To solve this issue, more appropriate systems have been developed to improve the stability of osteosynthesis systems, such as fixation with fixed angle plates and screws, blade plates, intramedullary rods, percutaneous pinning, tension band wires, and arthroplasty. ^(^ ^1^ Among the implants available for therapeutic application, special plates with locking screws seem to have a higher mechanical stability, and several clinical studies showed favorable results regarding their function and healing. ^(^ ^1^

Complications from this type of injury, such as neurological damage, vascular involvement, pseudoarthrosis, and osteonecrosis (ON), are frequent. According to Neer, ^(^ ^3^ one of the most relevant is the ON of the proximal humerus, which is influenced by age, the degree of injury (according to different fracture classification systems), the quality of reduction, implant positioning, and the energy of the trauma. ^(^ ^3^ ^),(^ ^4^

As for outcomes, it is possible to determine the prognosis of this condition during the preoperative phase, and detecting it at this stage can affect the final result. This injury causes significant pain and, in many cases, responds poorly to non-surgical treatment. It corresponds to approximately 5% of the preoperative diagnosis of all performed shoulder arthroplasties. ^(^ ^4^ ^),(^ ^5^

In reviewing the literature, we refer to the work of Hertel et al., ^(^ ^6^ who developed a binary classification system (LEGO^®^) with 12 possible types of proximal humeral fractures, from which, predictors of humeral head ischemia were defined as follows: length of the metaphyseal head extension < 8 mm; deviation of the medial hinge > 2 mm; basic fracture pattern (anatomical neck or epiphyseal separation); angular displacement of the head over 45°; fractures in three or four parts; the amount of displacement of tuberosities greater than 1 cm; and glenohumeral dislocation. Fractures of the anatomical neck of the humerus are associated with a “medial hinge” injury and calcar injury with metaphyseal involvement > 8 mm show 97% ON rates.

The association between proximal humeral fracture and ON is of great interest as are the orthopedic literature is yet to definitively explain several of its aspects. Such an issue instigated this group to conduct research aimed at predicting the risk of ON according to Hertel et al. ^(^ ^6^ and Neer’s^3^ classifications for surgically treated proximal humeral fractures after at least one year of postoperative follow-up.

## METHODS

This study was sent to the Research Ethics Committee via *Plataforma Brasil* and approved under CAEE No. 51474921.6.0000.5625 and opinion No. 4.958.341.

This is a retrospective, cross-sectional, observational cohort study that evaluated surgically treated adult proximal humeral fractures. Data collection occurred from January 2018 to January 2021.

Patients were selected from a patient database at our institution. The International Classification of Diseases (ICD 10) code S42 (fracture of shoulder and upper arm) was used. The following inclusion and exclusion criteria were determined:

Inclusion criteria:

1. Adults aged from 18 to 90 years.
2. All genders.
3. Complete medical records.
4. Diagnosis radiographs with good technical quality.
5. Postoperative radiographs with good technical quality.
6. Postoperative follow-up of at least a year.
7. Patients with no previous history of fracture and/or ON of the proximal humerus.
8. Patients with no previous history of neoplasms or oncological surgeries of the proximal humerus.
9. Patients without chronic inflammatory diseases.
10. Patients who agreed to participate in this research and signed informed consent forms.

Exclusion criterion:

1. Patients who refused either to participate in this study or to sign informed consent forms.

Therefore, 164 patients with proximal humeral fractures were found, of which 120 (73.17%) underwent conservative treatment and 44 (26.83%), surgical treatment. Of the operated patients, 16 (36.36%) were men and 28 (63.63%) were women, with an average age of 61.36 years.

All fractures were categorized according to the Neer classification. ^(^ ^3^ For each case, the chance of ON occurrence was considered positive for types 3 and 4.

The same cases were classified according to Hertel et al. ^(^ ^6^, and the prognosis of ON occurrence was considered on a case-by-case basis to determine the situations in which it could occur.

In the postoperative period with at least a year of follow-up, patients were evaluated for the presence or absence of ON. At this stage, our evaluation aimed to verify which had greater value to predict ON occurrence.

### Statistical analysis

Data were analyzed by a professional specialized in medical statistics using the IBM SPSS Statistics^R^, version 21, and Microsoft Excel^®^ 2010 (Microsoft Corporation^®^, San Diego, USA). Categorical data were shown as frequencies and percentages and continuous numerical data, as sample means and standard deviations. The Student’s t- (for normally distributed continuous numerical data from independent samples), Chi-square test (for categorical data), and Fisher’s exact tests (for cases that failed to meet Chi-square criteria) were applied. Analyses were performed to characterize the sample and identify the significant variables against the ON outcome, with statistical significance of 5%. A 2 × 2 table was used to evaluate the accuracy of selection and exactness of the classifications in Hertel et al. ^(^ ^6^ and Neer^3^ regarding ON. Cramer’s V coefficient was used to evaluate the association between variables in non-square tables.

## RESULTS

Of the patients in our series, three (6.8%) developed ON. We compared the Neer^3^ and Hertel et al. ^(^ ^6^ classifications with ON positive and negative outcome groups based on a 5% significance level. Both comparisons showed no statistically significant difference: p = 0.467 for the Neer classification^3^ (Table 1) and p = 0.177 for that of Hertel et al. ^(^ ^6^ (Table 2).

**Table 1:** Distribution of patients according to the Neer classification and the outcome regarding the presence or absence of osteonecrosis.

		Neer classification			p-value*
		1 part N (%)	2 parts N (%)	3 parts N (%)	
Osteonecrosis	No	10 (22.7)	26 (59.1)	5 (11.4)	0.467
	Yes	0 (0)	2 (4.5)	1 (2.3)	
	Total	10 (22.7)	28 (63.6)	6 (13.6)	

* Fisher’s Exact Test.

Cramer’s V 19.4%, p = 0.438.

**Table 2:** Distribution of patients according to the Hertel et al. classification and the outcome regarding the presence or absence of osteonecrosis.

		Criteria according to the Hertel et al. classification				p-value*
		0 N (%)	1 N (%)	2 N (%)	3 N (%)	
Osteonecrosis	No	10 (22.7)	16 (36.4)	14 (31.8)	1 (2.3)	0.177
	Yes	0 (0)	1 (2.3)	1 (2.3)	1 (2.3)	
	Total	10 (22.7)	17 (38.6)	15 (34.1)	2 (4.5)	

* Fisher’s Exact Test.

Cramer’s V 38.8%, p = 0.085.

Following the Neer classification, ^(^ ^3^ we classified 10 fractures (22.7%) as type 1; 28 (63.6%), as type 2; six (13.6%), as type 3; and none (0.0%), as type 4. In this group, three cases (6.8%) developed ON, two (4.5%) of type 2 and one (2.3%) of type 3. The p-value (0.467) showed no statistically significant correlation when we compared the presence or absence of ON using this classification system.

By the Hertel et al. ^(^ ^6^ classification, 10 fractures (22.7%) failed to meet criteria for ON risk, 17 (38.6 %) met one criterion, 15 (34.1%) met two criteria, and two (4.5%) met three criteria. The p-value (0.177) showed a higher correlation for Hertel et al. ^(^ ^6^ than Neer. ^(^ ^3^ However, our comparison with the positive outcome showed no statistical significance since one case (2.3%) had a positive outcome for ON and met one criterion for clinical risk according to the Hertel et al. classification, ^(^ ^6^ one (2.3%) met two criteria, and one (2.3%) met three criteria. The Neer classification^3^ showed a 19.4% Cramer’s V coefficient and Hertel et al. ^(^ ^6^, 38.8% with p = 0.438 and p = 0.085, respectively. This indicates that both classifications had no statistically positive relationship when compared to the development of ON. However, we observed a higher association for the Hertel et al. ^(^ ^6^ classification than that of Neer, ^(^ ^3^ although without statistical representation.

To evaluate the clinical applicability and predictive value of ON outcome of the evaluated classifications, we used 2 × 2 tables for high and low clinical risk: for the Neer classification, ^(^ ^3^ we considered fractures in one and two parts as low risk and in three parts as high risk; for the Hertel et al. ^(^ ^6^ classification, we considered fractures that met no or a criterion as low risk, and those that met two or three, as high risk for ON.

The Neer classification^3^ (Table 3) showed a 33.33% sensitivity, an 87.80% specificity, a 16.66% positive predictive value, and a 94.73% negative predictive value, whereas the Hertel et al. ^(^ ^6^ classification (Table 4), a 66.66% sensitivity, a 63.41% specificity, a 11.76% PPV, and a 96.29% NPV, indicating that both tools may be more useful to identify cases with low clinical risk and predict cases that are unable to develop into a negative outcome. When we compare both tools with our sample, the Neer^3^ classification showed higher specificity and that of Hertel et al. ^(^ ^6^, higher sensitivity. Both showed similar NPV.

**Table 3:** Cross table between high and low clinical risk for an osteonecrosis diagnosis according to the Neer classification.

		Osteonecrosis		Total
		Yes	No	
Neer	High risk	1	5	6
	Low risk	2	36	38
	Total	3	41	44

Fisher’s exact test, p = 0.363.

Low risk: fracture in one or two parts; High risk: fracture in three parts.

Sensitivity = 1/3 = 33.33%; Specificity = 36/41 = 87.80%; PPV = 1/6 = 16.66%; NPV = 36/38 = 94.73%.

**Table 4:** Cross table between high and low clinical risk for an osteonecrosis diagnosis according to the Neer classification.

		Osteonecrosis		Total
		Yes	No	
Hertel	High risk	2	15	17
	Low risk	1	26	27
	Total	3	41	44

Fisher’s exact test, p = 0.549.

Low risk: 0 or 1 criteria; High risk: 2 or 3 criteria.

Sensitivity = 2/3 = 66.66%; Specificity = 26/41 = 63.41%; PPV = 2/17 = 11.76%; NPV = 26/27 = 96.29%.

## DISCUSSION

Understanding the circulatory anatomy of the humeral head is key to justifying the important prevalence of ON of the humeral head, specifically after three or four parts are affected. However, this condition may have its genesis in more simplified fractures. ^(^ ^4^ ^),(^ ^7^

Some studies show that unfavorable functional outcomes are frequent considering displaced proximal humeral fractures with a concomitant diagnosis of ON. ^(^ ^8^ ^),(^ ^9^

In the early stages of this process, patients are usually asymptomatic or oligosymptomatic. Arthrofibrosis and pain progressively worsen. To minimize these impacts, these fractures should undergo anatomical reduction, which would offer satisfactory results. ^(^ ^4^ ^),(^ ^10^ Surgical treatment fails to provide superior results than conservative methods, ^(^ ^11^ ^),(^ ^12^ but arthroplasty is specifically used for patients with progressive symptoms, showing better outcomes.

However, other studies showed no better outcomes for the use of prosthesis than the surgical results of open reduction with osteosynthesis and hemiarthroplasty. ^(^ ^11^ ^),(^ ^12^

Considering older adults, primary hemiarthroplasty seems to determine more favorable results than using it as a therapeutic alternative for ON after osteosynthesis. ^(^ ^9^

The Neer^3^ classification system is widely known and often applied in patients with proximal humeral fractures. It states that a higher number of parts of the proximal portion determines the worst outcomes. Thus, stable reduction and osteosynthesis should be the preferred method for three-part displaced fractures and hemiarthroplasty for four-part ones. In the latter group, the method would directly relate to a greater chance of developing NO. ^(^ ^13^ ^),(^ ^14^

Our study used the classification of this author to assess if its application would be reliable for ON prognoses, as per Neer. ^(^ ^3^ However, we found a better tendency for the Hertel et al. ^(^ ^6^ classification, which statistically unproven.

Our study suggests that the criteria to determine ischemia in Hertel et al. ^(^ ^6^ may be useful to in safely anticipate NO prognoses orthopedists’ daily practice. However, our small sample size prohibited a statistically significant evaluation. Proper preoperative planning should also help in choosing the best therapeutic method and osteosynthesis device.

In 2015, Siebenbürger et al. ^(^ ^15^ investigated the moment of surgery and overall complication rates in the surgical treatment of proximal humeral fractures. After analyzing 329 patients (most of classified as having two- and three-part fractures), the authors found that surgery between 48 hours to five days failed to reduce complication rates. However, surgeries performed after that period significantly decreased complication rates, such as loss of reduction, loosening of synthesis material, and ON. They noticed an ON rate of 6.4% in their cases, resembling our findings.

According to the literature, the rates of this complication after surgical reconstruction of the humeral head range from 4 to 33%. Early stable internal fixation promotes humeral head revascularization by reducing ischemia time, which would decrease ON rates. ^(^ ^15^ ^),(^ ^16^

The glenohumeral dislocation associated with the fracture intensifies the circulatory severity of the proximal humerus as the displaced fragment has circulatory insufficiency due to the direct injury of the nutrient arteries of this segment and scarce soft tissue insertion. ^(^ ^17^ As most fracture-dislocations occur in a younger population, orthopedic surgeons should make a major effort to restore congruence to these injuries. We consider that lower pressure determined by locked plates, the concept of biological fixation, the use of minimally invasive techniques, indirect reductions, age below 50 years, and anatomical and stable reductions provide a substantial effect in protecting the vascular supply of the fractured humeral head.

Poor reduction and malunion seem to predispose the onset of ON. However, more research is needed to determine the causality of the relation between these factors. By analyzing the three cases of ON in our study in isolation, we observed that the poor quality of the reduction was an irrelevant factor.

This study has some limitations. Its small sample size (44 patients) and age heterogeneity (SD = 19.44) may have influenced our analysis of the correlations between the classifications to predict the outcome of interest after one year of follow-up.

## CONCLUSIONS

The Neer and Hertel et al. classifications show a similar discriminative ability to identify patients at low risk for developing ON (p = 0.467 for the Neer classification and p = 0.177 for the Hertel et al. classification).

New studies with a larger number of participants and greater sample homogeneity should increase the value of evaluations of the clinical applicability and predictive capacity of the studied classifications, with greater significance and correlation considering the ON outcome. Its validity should be considered as an important support tool in the daily practice of orthopedists.
